# Gene transfer of Hodgkin cell lines via multivalent anti-CD30 scFv displaying bacteriophage

**DOI:** 10.1186/1471-2199-9-37

**Published:** 2008-04-16

**Authors:** Yoon-Suk A Chung, Katja Sabel, Martin Krönke, Alexander Klimka

**Affiliations:** 1 University of Cologne, Institute of Medical Microbiology, Immunology and Hygiene, Goldenfelsstr. 19-21, 50935 Cologne, Germany

## Abstract

**Background:**

The display of binding ligands, such as recombinant antibody fragments, on the surface of filamentous phage makes it possible to specifically attach these phage particles to target cells. After uptake of the phage, their internal single-stranded DNA is processed by the host cell, which allows transient expression of an encoded eukaryotic gene cassette. This opens the possibility to use bacteriophage as vectors for targeted gene therapy, although the transduction efficiency is very low.

**Results:**

Here we demonstrate the display of an anti-CD30 single chain variable fragment fused to the major coat protein pVIII on the surface of bacteriophage. These phage particles showed an improved binding and transduction efficiency of CD30 positive Hodgkin-lymphoma cells, compared to bacteriophage with the anti-CD30 single chain variable fragment fused to the minor coat protein pIII.

**Conclusion:**

We can conclude from the results that the postulated multivalency of the anti-CD30-pVIII displaying bacteriophage combined with disseminated display of the anti-CD30 scFv on the whole particle surface is responsible for the improved gene transfer rate. These results mark an important step towards the use of phage particles as a cheap and safe gene transfer vehicle for the gene delivery of the desired target cells via their specific surface receptors.

## Background

The specific *in *or *ex vivo *transduction of cancerous, infected or malfunctioning cells with pro-apoptotic, defence or restoring genes is a promising approach for the treatment of patients. However, gene therapy continues to present obstacles, regarding safety, transduction efficiency and specificity. Most gene delivery systems are based on animal viral vectors, which are efficient on one hand, but may include the risk of intrinsic toxicity and are costly to manufacture. In addition to developing safer viral vectors, scientists began modifying bacteriophage particles for gene delivery purposes [1,2]. For example, filamentous bacteriophage libraries, consist of up to 10^10 ^unique phage particles; each displaying a different binding ligand on its surface and encapsulating the corresponding eukaryotic cDNA. These libraries are commonly used for the selection of novel antigen-specific binding molecules[3]. Furthermore, it is possible to select for ligand-bound phage being internalised by the target cell after receptor-binding [4,5]. If a eukaryotic gene cassette is encoded in the phagemid vector, a small percentage of target cells will express this gene [2,6,7]. Thus, bacteriophage have the potential to serve as specifically targeted gene delivery vectors. They have no natural tropism for eukaryotic cells, nor do they integrate into the cell's genome.

A filamentous bacteriophage containing a phagemid vector (herein after referred to as "phagemid particle") encodes only the gene for a surface fusion protein, an antibiotic resistance gene and an amplification and packaging signal, but lacks the genes for all other bacteriophage proteins. In order to produce functional phagemid particles, *E. coli *transformed with the phagemid vector have to be superinfected with helper phage (phage rescue), which contribute the missing proteins for the phagemid particle assembly in the periplasm of *E. coli*. Phagemid particles are easy to genetically modify in terms of targeting to a specific receptor-bearing cell population. As phagemid vectors are relatively small in size compared to full size phage vectors, they are capable of carrying larger foreign DNA sequences. However, a major obstacle of using bacteriophage as gene transfer vehicles is their low transduction efficiency, which relies on gene transfer and expression. Gene transfer efficiency generally depends on the binding affinity of the ligand and the intracellular trafficking of the internalized receptor-bound phage, including endosomal escape and capsid breakdown. Moreover, processing of the single-stranded phage DNA, including conversion into double-stranded DNA and nuclear localization, is a critical factor [8].

Commonly, the displayed ligand is fused to the minor coat protein pIII (5 copies per phage). Ligands can be displayed either monovalently or multivalently, depending on the helper phage system being used. The importance of this factor has been demonstrated by Larocca et al. (2001) [9] in comparing the transduction efficiency of epidermal growth factor receptor (EGFR)-positive target cells with bacteriophage containing a green fluorescence protein (GFP) gene and displaying either monovalently or multivalently the EGF molecule. The multivalency of the EGF, fused to the minor coat protein pIII on the phage particles, increased transduction efficiency by 5-fold. Similar effects have been shown by Poul and Marks (1999) using a pIII-displayed anti-ErbB2 scFv.

Moreover, it has been demonstrated that phages displaying ligands fused to their major coat protein pVIII (2700 copies per phage) are able to bind to cell surface receptors, be internalized and processed inside mammalian cells [10] leading to marker gene expression [7]. It is generally believed that the wild type to fusion protein ratio of pVIII displaying phage is inversely related to the size of the displayed ligand, making it difficult to functionally express larger proteins on the phage surface [11]. Nevertheless, it has been shown that this correlation is not so stringent. Malik *et al*. (1996) demonstrated that Fab fragments and enzymes can be displayed on pVIII, but with a frequency of less than 1% [12].

We were interested in developing a gene delivery vector for the specific transduction of Hodgkin/Reed-Sternberg cells, the malignant cell type of Hodgkin's lymphoma. The CD30 receptor has been exploited as Hodgkin-lymphoma associated antigen in recent experimental therapeutic strategies [13,14]. Antibodies bound to CD30 are internalized by clathrin-mediated endocytosis and traffic to the lysosome [15]. Regarding this internal trafficking of CD30-bound complexes as a prerequisite for gene transfer, we generated a phagemid vector that encodes an anti-CD30 scFv-pVIII fusion protein and a eukaryotic CMV-EGFP gene cassette. Out of this vector we produced small, anti-CD30 phagemid particles, capable of delivering EGFP as a reporter gene for transduction. We chose the major coat protein pVIII as the fusion partner for the display of the anti-CD30 scFv Ki-4 [14]. Fusion with pVIII as an alternative to the widely used minor coat protein pIII should increase the multivalency of the scFv-display and demonstrate the feasibility of displaying relatively big molecules, like scFvs (27 kDa) on pVIII.

## Results

### Construction of the anti-CD30 gene transfer phagemid vectors

**Figure 1:**
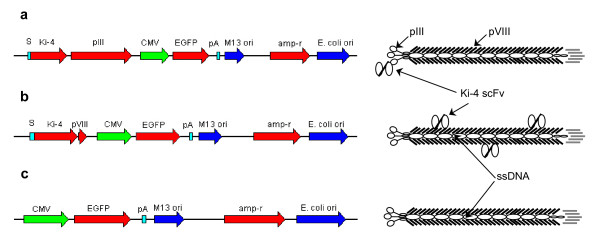
**Phagemid vectors pCANTAB6-Ki-4(EGFP), pAK8-Ki-4(EGFP) and pAK8(EGFP)**. Schematic drawing of phagemid vectors and deduced phagemid particles used for gene transfer. pCANTAB6-Ki-4(EGFP) (**a**) and pAK8-Ki-4(EGFP) (**b**) contain the pIII signal sequence (S) adjacent to the Ki-4 scFv gene. pCANTAB6-Ki-4(EGFP), pAK8-Ki-4(EGFP) and pAK8(EGFP) (**c**) contain the enhanced green fluorescence protein gene (EGFP), under the control of a cytomegalovirus promoter (CMV) and terminated by a poly adenylation site (pA), as well as an M13 origin of replication site, with a DNA packaging signal sequence (M13 ori), an ampicillin resistance gene (amp-r) and an *E. coli *origin of replication site (E. coli ori).

The anti-CD30 scFv Ki-4 has been selected from a Ki-4 hybridoma-derived phage display library via cell panning on L540 Hodgkin cells as previously described [14]. The eukaryotic CMV-EGFP gene cassette was excised from the pEGFP-N2 expression vector and ligated into the phagemid vector pCANTAB6-Ki-4, resulting in the vector pC6-Ki-4(EGFP). Transformation of *E. coli *TG-1 and subsequent packaging of the pC6-Ki-4(EGFP) phagemid vector with M13 helper phage leads to M13-pC6-Ki-4(EGFP) phagemid particles with the Ki-4 scFv monovalently displayed as a fusion protein with the minor coat protein pIII (Fig. 1a). An exchange of the pIII gene of pC6-Ki-4 with the pVIII gene from the M13 genome and the insertion of the CMV-EGFP gene cassette, resulted in the phagemid vector pAK8-Ki-4(EGFP). Rescuing of phagemid particles with this phagemid vector will produce the M13-pAK8-Ki-4(EGFP) phagemid particles with Ki-4 scFv displayed in multiple copies on their surface (Fig. 1b). As a control, the Ki-4 scFv gene was deleted from the pAK8-Ki-4(EGFP) phagemid vector, to produce the phagemid particles M13-pAK8(EGFP) displaying no anti-CD30 scFv on their surface, but containing a functional eukaryotic EGFP expression cassette (Fig. 1c).

### Detection of Ki-4 scFv fused to bacteriophage coat protein pVIII

**Figure 2:**
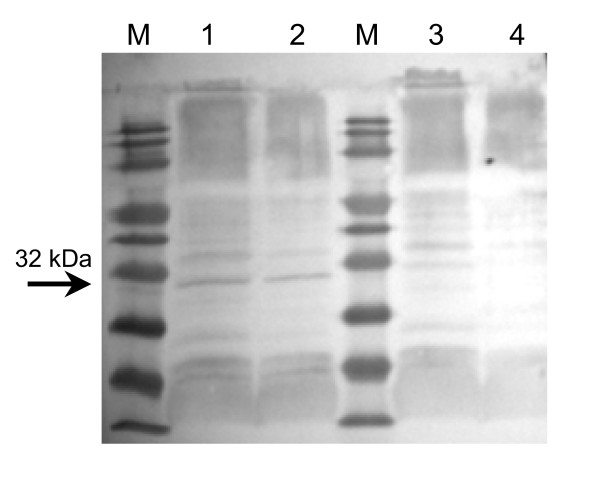
**Detection of displayed Ki-4 scFv fusion protein**. Western blot analysis of 8 × 10^10 ^(lanes 1 and 3) or 4 × 10^10 ^(lanes 2 and 4) of purified phagemid particles M13-pAK8-Ki-4(EGFP) (lanes 1 and 2) or M13-pAK8(EGFP) (lanes 3 and 4), respectively. The fusion protein was detected using anti-M13-pVIII- and goat-anti-mouse-HRP-antibodies. Arrow indicates pVIII-scFv fusion protein.

After superinfection of the phagemid vector containing *E. coli *clones with helper phage, the phagemid particles assemble in the bacterial periplasm and package the phagemid single-stranded DNA with the major coat protein (pVIII) and the minor coat proteins (pIII, pVI, pVII and pIX). During this process, the N-terminal signal sequences of the coat proteins pIII and pVIII are cleaved leaving the N-terminal part of these proteins outside of the bacteriophage capsid and accessible for binding interactions. The predicted size of the major coat protein pVIII (5 kDa) fused with the Ki-4 scFv (27 kDa) is 32 kDa (Ki-4-pVIII). In Western blot analysis this fusion protein was detected in M13-pAK8-Ki-4(EGFP) and not in the control M13-pAK8(EGFP) phagemid particles (Fig. 2).

### Specific CD30 binding of Ki-4 scFv-displaying phage

**Figure 3:**
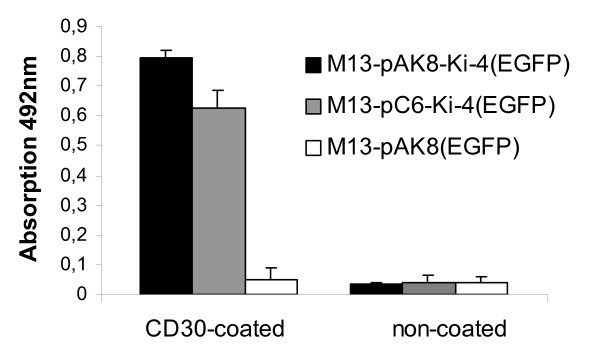
**Specific anti-CD30 binding of Ki-4 scFv-displaying phagemid particles**. CD30 antigen (50 ng/ml) was coated to ELISA MaxiSorp plates and 4 × 10^10 ^phagemid particles were added, respectively. Bound phagemid particles were detected using anti-M13-pVIII-HRP-antibody.

To demonstrate that phagemid particles displaying the Ki-4 scFv specifically bind CD30, an enzyme-linked immuno sorbent assay (ELISA) was performed, coating wells with recombinant CD30 and incubating them with 4 × 10^10 ^phagemid particles. A strong interaction of the anti-CD30 Ki-4 scFv-displaying phagemid particles M13-pAK8-Ki-4(EGFP) and M13-pC6-Ki-4(EGFP) with the coated CD30 was detected. The control phagemid particle M13-pAK8(EGFP) and phagemid particles incubated in non-coated wells did not bind (Fig. 3). The binding of M13-pAK8-Ki-4(EGFP) to the CD30 antigen was stronger than the binding of the M13-pC6-Ki-4(EGFP).

**Figure 4:**
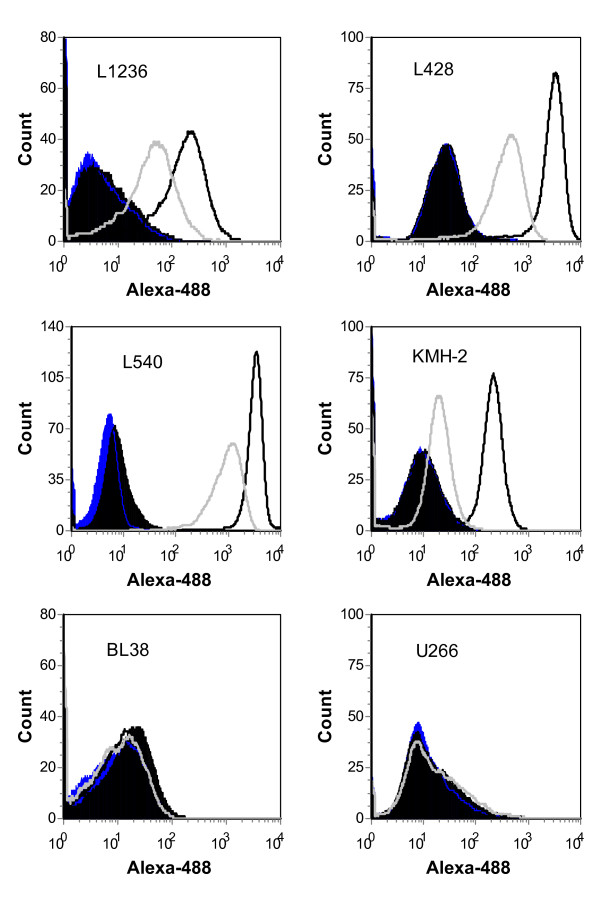
**Cell binding studies of M13-pAK8-Ki-4(EGFP), M13-pC6-Ki-4(EGFP) and M13-pAK8(EGFP) phagemid particles**. 2,5 × 10^5 ^of indicated cells (filled black) were incubated with 2 × 10^12 ^phagemid particles (M13-pAK8-Ki-4(EGFP) black line, M13-pC6-Ki-4(EGFP) gray line or M13-pAK8(EGFP) dark gray line) and subsequently incubated with mouse anti-M13 IgG and Alexa Fluor 488-conjugated anti-mouse IgG antibody. Mean fluorescence intensity of cells was analyzed by fluorescence-activated cell sorting (FACS).

To demonstrate the cell binding specificity of the anti-CD30 phagemid particles, FACS analyses were performed. Confirming the ELISA results, the Ki-4 scFv displaying phagemid particles bound only to the CD30-positive, Hodgkin-derived cell lines L1236, L540, L428 and KMH-2 and not to the CD30-negative multiple myeloma cell line U266 and the Burkitt Lymphoma cell line BL38. The control phagemid particle M13-pAK8(EGFP) did not show binding to any cell line. Furthermore, the FACS analysis revealed that M13-pAK8-Ki-4(EGFP) binding to the target cells is consistently stronger than the binding of the M13-pC6-Ki-4(EGFP) phagemid particles (Fig. 4).

**Figure 5:**
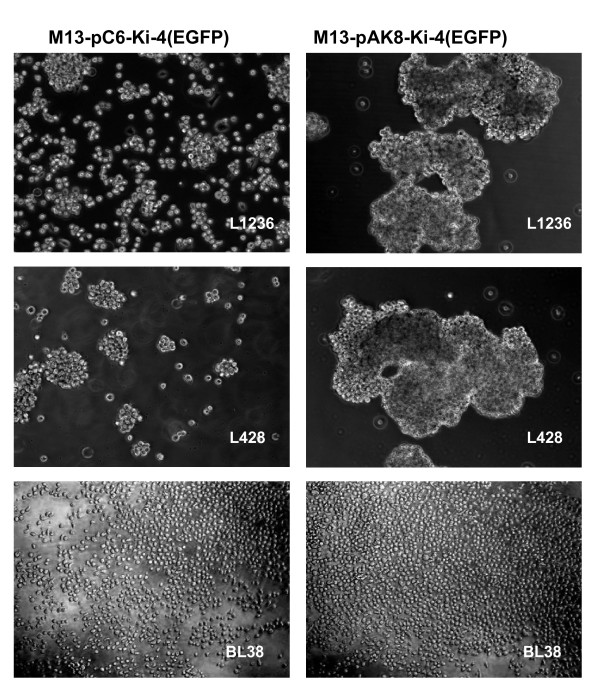
**Cluster formation of CD30+ cells incubated with M13-pAK8-Ki-4(EGFP)**. 5 × 10^4 ^L1236, L428 and CD30-negative BL38 cells were incubated with 5 × 10^11 ^M13-pC6-Ki-4(EGFP) or M13-pAK8-Ki-4(EGFP) phagemid particles for 24 hrs, respectively. Representative sections were photographed with 10 × objective.

The incubation of the CD30-positive cells with the anti-CD30 scFv-displaying phagemid particle M13-pAK8-Ki-4(EGFP) leads to additional cluster formation of the cells after 24 hours, in contrast to the M13-pC6-Ki-4(EGFP) phagemid particles (Fig. 5). No cluster formation was seen with CD30-negative cells incubated with M13-pAK8-Ki-4(EGFP).

### Specific gene transfer of CD30-positive cells by targeted bacteriophage

**Figure 6:**
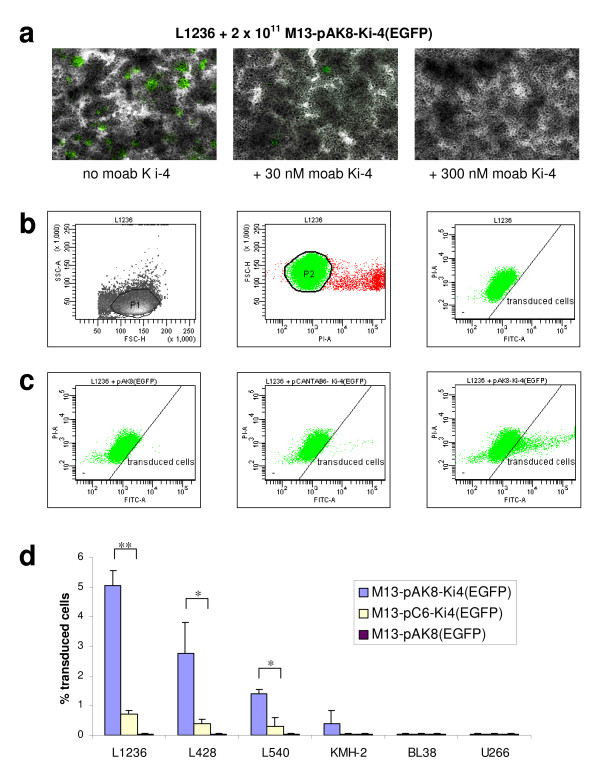
**Specific gene delivery of CD30 positive cell lines**. Gene delivery specificity was demonstrated via competition experiment. (**a**) L1236 cells were incubated with M13-pAK8-Ki-4(EGFP) phagemid particles in the absence or presence of monoclonal antibody (moab) Ki-4. Representative sections were photographed with 10 × objective using fluorescence microscopy. Transduction efficiency was quantified by FACS analysis after 96 hours. Indicated cell lines were incubated with M13-pAK8-Ki4(EGFP), M13-pCANTAB-Ki4(EGFP) or M13-pAK8(EGFP) phagemid particles, respectively. (**b**) A homogenous cell population (P1) was gated in forward (FSC-H) by right-angle light (SSC-H) scatter plots and subsequently gated for living cells (P2) in FSC-H by propidium iodide (PI-A) channel dot plot. The living cells were finally analyzed for EGFP-expression in PI-A versus green fluorescence (FITC-A) channel dot plot, setting the gate at 0.05% of transduced cells incubated with the control phagemid particles M13-pAK8(EGFP). (**c**) Representative analysis of the L1236 cell line, incubated with M13-pAK8(EGFP), M13-pCANTAB6-Ki4(EGFP) or M13-pAK8-Ki4(EGFP) phagemid particles, respectively. Cells were analyzed for EGFP expression as described above. (**d**) Summarized results of transduction experiments with different cell lines in percentage of EGFP-expressing cells. Statistical significance was analysed according to the Student's one-tailed *t *test. *p*-values < 0.05, < 0.01 and < 0.001 were indicated with *, ** or ***, respectively.

We performed competition assays to determine if CD30-binding phagemid particles are capable of antigen-specific gene delivery of a EGFP reporter gene. 5 × 10^4 ^CD30-positive L1236 cells were incubated with 2 × 10^11 ^M13-pAK8-Ki-4(EGFP) phagemid particles in the absence or presence of 30 nM or 300 nM of the parental monoclonal antibody Ki-4, respectively (Fig. 6a). In the absence of the competitor about 0.1% of the cells show EGFP expression demonstrating gene transfer. In the presence of the parental monoclonal antibody Ki-4, the number of transduced cells is clearly reduced in a dose dependent manner, proving the antigen-specificity of the gene transfer. To further demonstrate antigen-specificity and evaluate transduction efficiency, 5 × 10^4 ^of the CD30-positive L1236, L428, L540 and KMH-2 cells or the CD30-negative BL38 or U266 cell lines were incubated for 96 hours with 2 × 10^12 ^phagemid particles M13-pAK8(EGFP), M13-pAK8-Ki-4(EGFP) or M13-pC6-Ki-4(EGFP), respectively. The percentage of living cells expressing the eukaryotic gene cassette CMV-EGFP was determined by FACS analysis after propidium iodide staining as depicted in Fig. 6b. As shown in Fig. 6c the L1236 cells remain untransduced when incubated with the control phagemid particles M13-pAK8(EGFP). Phagemid particles displaying the Ki-4 scFv fused to the coat protein pIII (M13-pC6-Ki-4(EGFP)) minimally transduced the L1236 cells (0.8%). In contrast, L1236 cells incubated with the same amount of M13-pAK8-Ki-4(EGFP) phagemid particles displaying Ki-4 scFv-pVIII fusion proteins, the transduction efficiency was significantly increased to approximately 5%. The superior transduction efficiency of the M13-pAK8-Ki-4(EGFP) phagemid particles was also seen with the other CD30-positive cell lines, as summarized in Fig. 6d. No transduction could be seen with the CD30-negative cell lines BL38 or U266 using these phagemid particles.

## Discussion and Conclusion

Use of bacteriophage as vectors for targeted gene transfer into eukaryotic cells is an alternative method to circumvent some of the problems occurring with the established viral vectors. Bacteriophage have no natural tropism for eukaryotic cells but can be targeted towards specific cell surface receptors by displaying appropriate binding ligands, such as peptides or antibody fragments. Internalized by receptor-mediated endocytosis, their packaged single-stranded DNA is further processed (mechanisms not fully understood), leading to the expression of encoded genes under eukaryotic promoters [6]. The ease with which one can target, modify, produce and purify these phage makes them a valuable tool for antigen-specific cell transduction. Here, we describe the generation of anti-CD30 targeted phagemid particles, specifically binding and transducing CD30-positive cells. For this purpose, the anti-CD30 Ki-4 scFv gene has been genetically fused either to the major coat protein pVIII (pAK8-Ki-4(EGFP)) or the minor coat protein pIII (pC6-Ki-4(EGFP)) in a phagemid vector, which also encodes an EGFP gene under the control of a CMV promoter. The expression of the Ki-4 scFv-pVIII fusion protein was determined by Western blot analysis (Fig. 2) and ELISA experiments with recombinant CD30 antigen demonstrating the specific binding of the Ki-4 scFv displaying phagemid particles (Fig. 3). Additionally, phage displaying the Ki-4 scFv showed a specific binding to CD30-bearing cells in FACS analyses. Binding affinity of the M13-pAK8-Ki-4(EGFP) phagemid particles to CD30 antigen and CD30 receptor-bearing cells was always higher, compared to the M13-pC6-Ki-4(EGFP) phagemid particles. Provided that scFvs fused to the pIII coat protein have the same binding affinity as pVIII-fusion proteins, this difference could be explained by a multivalent display of the anti-CD30 Ki-4 scFv on the M13-pAK8-Ki-4(EGFP) phagemid particles indicating a binding avidity effect. The multivalent display of the scFv on the M13-pAK8-Ki-4(EGFP) phagemid particles is also supported by the fact that these phagemid particles are able to induce additional cluster formation of CD30-positive cells (Fig. 5). This phenomena was not seen with the supplement of M13-pC6-Ki-4(EGFP) phagemid particles or the addition of the M13-pAK8-Ki-4(EGFP) phagemid particles to the CD30-negative cell line BL38, but has been observed previously by the addition of monoclonal Ki-4 IgG antibody to CD30-positive cells (data not shown).

The transduction experiments with different cell lines revealed that anti-CD30 scFv-displaying phagemid particles are able to transduce CD30 receptor-bearing cells. The addition of the parental monoclonal antibody Ki-4 significantly reduced the amount of CD30-positive L1236 cells transduced by the M13-pAK8-Ki-4(EGFP) phagemid particles (Fig. 6a). There was no unspecific transduction of CD30-negative cells, nor did control phagemid particles M13-pAK8(EGFP) transduce the CD30-positive cells (Fig. 6d). This indicates that CD30 antigen on the targeting cell lines is required for transduction with anti-CD30 phagemid particles and establishes the high specificity of these gene transfer particles. Incubation of the target cells with the M13-pAK8-Ki-4(EGFP) phagemid particles led to a maximum transduction rate of 5%, a rate approximately 10-fold higher compared to the M13-pC6-Ki-4(EGFP) phagemid particles. This enabled a direct comparison of pVIII- and pIII- fusion protein display for receptor-specific gene transfer. Although there is a postulated avidity effect responsible for the stronger binding of the M13-pAK8-Ki-4(EGFP) phagemid particles to recombinant CD30 and CD30-positive cells compared to the M13-pC6-Ki-4(EGFP) phagemid particles, this significant difference in transduction efficiency was somewhat surprising. Other groups using monovalent pIII-display with transduction efficiencies of 1–4% were able to increase the efficiencies up to 10% by using multivalent pIII-display [6,9].

It has been described that CD30 receptor is being internalized by clathrin-mediated endocytosis, maturing to lysosomes and that cross-linking of CD30 receptors by monoclonal antibodies enhances this trafficking at least by 3-fold [15]. In accordance with our results, this supports our hypothesis that pVIII-based display of scFvs is multivalent, leading to increased internalization and transduction efficiency of CD30-bearing cells. Although it has been shown that multivalent scFv-pIII display on bacteriophage is possible and increases transduction efficiency [9], our approach serves as an alternative or even superior system, especially if the internalization depends on multipoint 'zipper' contacts of the phage with the cell surface receptors. A multivalent display on pVIII may provide an increased binding and phage uptake because pVIII fusion proteins are spread over the surface of the phage, in contrast to pIII fusion proteins, which are focused on the tip of the phage. This so-called 'disseminated display' would also explain why M13-pAK8-Ki-4(EGFP), in contrast to the M13-pC6-Ki-4(EGFP) phagemid particles, are able to connect CD30-positive cells leading to cluster formation (Fig. 5). This also explains the stronger binding activity of the M13-pAK8-Ki-4(EGFP) phagemid particles (Fig. 4) and the higher transduction rate (Fig. 6d). However, this potential cross-linking of cells by single M13-pAK8-Ki-4(EGFP) phagemid particles does not prevent internalization of other M13-pAK8-Ki-4(EGFP) phagemid particles, because simultaneous binding of receptors on one cell is even more likely than cross linking of receptors on separate cells.

The feasibility of displaying molecules as large as scFvs (27 kDa) on the major coat protein raises the possibility of generating bispecific phagemid particles rescued from *E. coli*, encoding an additional pIII- or pVIII-fusion protein, thereby increasing specificity and avidity of the cell-targeting phagemid particle.

The unequal transduction rates of the different CD30-positive cell lines with the M13-pAK8-Ki-4(EGFP) phagemid particles are not directly explainable by their individual CD30 receptor densities. For example, cell line L1236 showed only a moderate binding of the anti-CD30 phagemid particles in FACS analysis compared to the L540 cell line(Fig. 4), but revealed the highest EGFP-expression rate (Fig. 6c). This demonstrates that the efficiency of the gene transfer seems to be dependent on cell line-specific trafficking of the bacteriophage after binding. Because the gene transfer efficiency of targeted bacteriophage is in general still very low, it seems to be necessary to further optimize trafficking, DNA processing and nuclear localization of the phagemid DNA. So far, satisfying transduction rates (30–45%) with bacteriophage have been reached only *in vitro *by genotoxic treatment [16].

To our knowledge, this is the first approach demonstrating a potential advantage of the major coat protein pVIII as fusion partner for the display of scFvs in gene transfer experiments, compared to the commonly used minor coat protein pIII. Furthermore, it is the first approach generating a CD30-specific gene transfer vector to be used for the transduction of Hodgkin-, Reed Sternberg cells. A possible application of this system would be the specific induction of apoptosis in these cells. It is believed that cancer cells are on the verge of apoptosis, containing processed effector caspases. At the same time, they also express inhibitor of apoptosis proteins (IAPs), which can not be neutralized due to malfunctioning apoptotic pathways [17]. It has been demonstrated that overexpression of second mitochondrial activator of caspases (SMAC) in Hodgkin-derived cell lines blocks X-linked IAPs activation [18], and therefore would make a promising candidate gene for restoration of the apoptotic pathway in Hodgkin's disease via targeted gene therapy. Provided that the transduction efficiency of CD30-specific bacteriophage is further increased by additional molecular modifications, they can potentially serve as specific, safe, cheap and efficient gene therapy vectors for *in *and *ex vivo *applications. This is supported by the fact that tumor treatment with targeted bacteriophage *in vivo *has been demonstrated recently [19].

## Methods

### Cells and Bacteria

The Morbus Hodgkin derived cell lines L540 [20], L1236 [21], L428 [22], KMH-2 [23], the Burkitt lymphoma cell line BL38 [24] and the multiple myeloma cell line U266 [25] were cultivated in RPMI 1640 supplemented with 10% (v/v) heat inactivated fetal calf serum (FCS), 50 μg/ml Penicillin, 100 μg/ml Streptomycin and 2 mM L-glutamine. All cells were cultured at 37°C in a 5% CO_2 _atmosphere. *E. coli *TG-1 (K12 Δ(*lac-pro*), *sup*E, *thi, hsd*D5F'*tra*D36, *proA+B+*, *lacI*q, *lacZ*ΔM15) were provided by CAT (Cambridge, UK).

### Plasmids

pAK8 was cloned by exchanging the minor coat protein pIII gene from pCANTAB6-Ki-4 phagemid vector [14] with the major coat protein pVIII gene from KM13-K07 helper phage genome. pVIII was amplified by PCR using the primer pair pVIII NotI sense 5'-ATC GCG GCC GCA GAG GGT GAC CCC-3' and pVIII EcoRI antisense 5'-ACT GAA TTC TTA TCA GCT TGC TTT-3'. The PCR product was digested with *Not*I/*Eco*RI and ligated into pCANTAB6-Ki4 phagemid vector. pAK8-Ki-4 and pCANTAB6-Ki-4 phagemid vectors were digested with restriction enzyme *EheI *creating blunt ends and ligated with the reporter gene cassette CMV-EGFP, obtained from pEGFP-N3 plasmid (Clontech, USA) that had been digested with *VspI/BspT1 *and treated with T4 DNA polymerase to produce blunt ends. The resulting phagemid vectors were pAK8-Ki-4(EGFP) and pC6-Ki-4(EGFP). The control phagemid vector pAK8(EGFP) was constructed by excising the Ki-4 scFv gene via *Pvu*II digest.

### Phagemid particle preparation and purification

For production of M13-pAK8-Ki4(EGFP), M13-pC6-Ki4(EGFP) and M13-pAK8(EGFP) phagemid particles, phagemid vector transformed overnight culture of *E. coli *TG-1 were grown and used to inoculate 50 ml 2xYT + 100 μg/ml ampicillin at a ratio of 1:100 at 37°C with vigorous shaking (220 rpm) to an OD_600 _of 0.5. 5 ml of the culture were infected with helper phage (KM13-K07) at a ratio of 4 phage per bacterium and incubated at 37°C in a water bath without shaking for 30 min and 30 min at 37°C with shaking (220 rpm). Bacteria were spun down, resuspended in 25 ml 2xYT + 100 μg/ml ampicillin + 50 μg/ml kanamycin and shaken overnight at 30°C. After removing of bacteria by centrifugation (20 min at 4400 g) supernatant was transferred into fresh bottles and phage were purified by two rounds of standard polyethylene glycol (PEG) precipitation as described [26]. The final phage pellet was resuspended in 1.5 ml PBS. Phage titers in colony forming units (cfu) were determined by photometrical absorption at 269 nm as described [27].

### ELISA

Multi-well Maxisorp plates (Nunc, Germany) were coated for 1 h at room temperature with 50 μl of recombinant CD30 antigen (R&D Systems, USA) at a concentration of 10 μg/ml in bicarbonate buffer, pH 9.6. After discarding the coating solution, wells were washed 5 × with 200 μl PBS-0.1% Tween and incubated for 1 h with blocking solution (PBS-4% skimmed milk powder). After washing, 50 μl of M13-pAK8-Ki-4(EGFP), M13-pC6-Ki-4(EGFP) and M13-pAK8(EGFP) phagemid particles (4 × 10^10 ^cfu) were added to the wells and allowed to bind for one hour. Plates were washed 10 × with 200 μl PBS-0.1% Tween and 50 μl of a 10 μg/ml solution of anti-M13-pVIII-HRP-conjugated antibody (Amersham, USA) in blocking buffer was added to each well and incubated for 1 h. Plate was washed and horseradish peroxidase activity was detected by incubation with 50 μl OPD-substrate (Pierce, USA) and reaction was stopped by adding 25 μl H_2_SO_4 _(1N). Substrate conversion was read by an automated ELISA reader and the results were expressed as *A *= *A*_492 nm_-*A*_620 nm_.

### SDS-PAGE and Western Blot Analysis

25 μl of purified phage samples (5 × 10^12 ^particles/ml) per lane were separated by 14% SDS-PAGE and transferred to a nitrocellulose membrane using a Mini Protean III Blotting unit (BioRad, Germany) at 250 mA for 90 min. Membrane was blocked with blocking buffer for 1 h, washed 3 × for 5 min with PBS, then anti-M13-pVIII antibody solution (1 μg/ml) in blocking buffer was added and incubated for 1 h at RT with gentle shaking. Membrane was washed 3 times and goat-anti-mouse-HRP-conjugated antibody in blocking buffer (1 μg/ml) was added followed by 1 h incubation at RT. After washing three times with PBS Western Blots were developed using the CN/DAB substrate kit (Pierce).

### FACS analysis of phagemid particles binding to cell lines

Cells (5 × 10^5^) were washed twice with 1 ml FACS wash buffer (PBS, 2% FCS, 0.1% NaN_3_) by centrifugation (300 g) and incubated with 5 × 10^9 ^phagemid particles in 100 μl for 1 h at 4°C. Cells were washed twice as described above and incubated with 100 ng murine anti-M13 antibody (Progen, Germany) in 100 μl. Cells were washed twice and incubated with 200 ng Alexa Fluor 488-conjugated goat anti-mouse antibody (Invitrogen, Germany) in 50 μl. Cells were washed and analyzed for green fluorescence using a Becton Dickinson FACScanto cytometer and Becton Dickinson FACSDiva software.

### Transduction assays

Cells were seeded at 5 × 10^4^/well in 12-well plates in complete medium and 500 μl phage suspension (4 × 10^12 ^phagemid particles/ml) was added to a total volume of 1.5 ml and incubated for 48 h. After 48 h 1 ml complete medium was added and cells were incubated for additional 48 h. EGFP expression was determined by flow cytometry. To determine the significance of the data, statistical analysis were performed according to the Student's one-tailed *t *test. *p*-values < 0.05, < 0.01 and < 0.001 were indicated with *, ** or ***, respectively. For the competition assay 5 × 10^4 ^L1236 cells per well were incubated with 2 × 10^11 ^M13-pAK8-Ki-4(EGFP) phagemid particles in the absence or presence of 30 nM or 300 nM of the parental monoclonal antibody Ki-4. After 96 hours cells were analyzed for EGFP expression by fluorescence microscopy.

## Authors' contributions

YSAC carried out most of the experiments, analyzed the results and helped to draft the manuscript. KS participated in the experiments and helped to draft the manuscript. MK contributed to the interpretation of the data and helped to draft the manuscript. AK conceived the study, designed and performed experiments and drafted the manuscript. All authors read and approved the final manuscript.
